# Oligocene incursion of the Paratethys seawater to the Junggar Basin, NW China: insight from multiple isotopic analysis of carbonate

**DOI:** 10.1038/s41598-020-63609-0

**Published:** 2020-04-20

**Authors:** Qian Li, Long Li, Yuanyuan Zhang, Zhaojie Guo

**Affiliations:** 1 Key Laboratory of Orogenic Belts and Crustal Evolution, School of Earth and Space Sciences, Peking University, Beijing, 100871 PR China; 2 Department of Earth and Atmospheric Sciences, University of Alberta, Edmonton, AB Canada

**Keywords:** Solid Earth sciences, Geochemistry, Sedimentology

## Abstract

The Cenozoic retreat of the Paratethys Sea, as well as uplift of the Tibet plateau and global cooling, have been considered as the main mechanisms for the onset of the Asian monsoon. However, due to the weak constraints on the time and path of the Paratethys Sea, the relative contribution of each of the three factors to the onset of the Asian monsoon remains debatable. Previous studies on the retreat process of the Paratethys suggested that its east coastline had already withdrawn from the Chinese Tianshan area to the west by the Oligocene. Here, we provide a new perspective on this discussion through the study of the Oligocene Anjihaihe dolomite from the northern Chinese Tianshan. Through the comparisons of carbon, oxygen, magnesium, and strontium isotope compositions between the dolomite beds and their interlayered precursor lacustrine limestone beds, we show that the Anjihaihe dolomites were formed at the end of the Oligocene from dolomitization by a fluid characterized by high δ^18^O, low δ^26^Mg, low temperature, relatively poor in carbon and Sr. This fluid was likely derived from seawater from the close-by Paratethys Sea at the time. This discovery suggests a larger Paratethys Sea during the Oligocene than previously thought and may have important implications to understand the evolution of the palaeogeography in the Chinese Tianshan region and the onset of the Asian monsoon.

## Introduction

The Cenozoic uplift of the Tibetan plateau and subsequent retreat of the proto-Paratethys Sea, as well as global cooling, are considered to be the major factors responsible for the origin of the Asian monsoons climate, although the detailed mechanism and relative contribution of each factor are still not clear^1–8^. To unravel the relative roles of these potential affecting factors and to understand the palaeogeographic evolution and climatic change in northwest China and central Asia, the retreat history of the proto-Paratethys Sea in the context of the uplift of the Tibetan plateau is a key.

Previous studies indicate that, by the Eocene–Oligocene boundary, the proto-Paratethys Sea has already been disconnected from its southern neighbour the Mediterranean Sea to form the ParatethysSea, due to the strong tectonic activities in the Eurasian Plate^9,10^. On its eastern side, the coastline of the Paratethys Sea started to withdraw from the Chinese Tianshan area to the west^9^, resulting in the loss of an important moisture source, increased land-sea thermal contrasts, and strengthened Asian monsoons^1,3,5,6^. In the Oligocene, the shallow epicontinental Paratethys Sea is believed to occasionally connect to its southern adjacent Mediterranean Sea in the west, depending on the open and closure of the seaway^5,6,10–15^; but its boundary in the east sidewas poorly constrained, although a general thought considers it has retreated to the west far away from the Chinese Tianshan Mountain^10,13^.

Recently, dolomites beds interlayered with beds of siltstone, mudstone or limestone were discovered in the Oligocene Anjihaihe Formation in the Junggar Basin in the north of the Chinese Tianshan Mountains. Petrographical examinations show that the dolomite occurs mainly as a dolomicrite with low-crystallinity^16^. Based on preliminary Mg isotope compositions of the dolomite beds, Li *et al*.^16^ speculate the dolomitization process for the formation of these dolomite beds could have involved seawater but did not provide a further demonstration. However, given that the siltstone, mudstone and limestone in the Anjihaihe Formation were all lacustrine in origin, the source and inflow pathway of the involved seawater is puzzling – whether it was older seawater that had been buried in deeper sedimentary layers but reactivated in the Oligocene by tectonic activities, or it was concurrent seawater that invaded into the Junggar Basin. In addition, whether the repeated layers of dolomite were produced by a single or repeated incursion of seawater is also an important question to address in order to understand the regional tectonic and environmental history.

In this contribution, we provide new strontium and magnesium isotopic data, together with the published but undiscussed carbon, oxygen, and magnesium isotopic data^16^ for the dolomite beds and their interlayered limestone beds, to show the dolomitization was induced by one episodic incursion of seawater, likely from the Paratethys Sea into the Junggar Basin in the Oligocene. The discovery of connections between the north Tianshan area and the Paratethys Sea in the Oligocene may have implications for the onset and development of the regional climate related to the Asian monsoon.

## Geological setting

**Figure 1:**
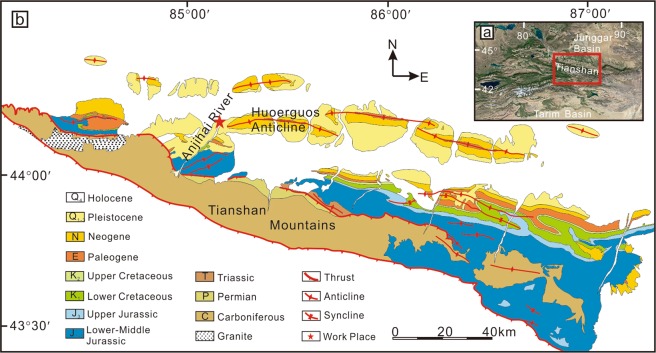
Geological background of northwest China. (**a**) The satellite image of northwest China, including the Junggar Basin, Tianshan Mountains, and Tarim Basin from Google Earth (map data: Google, Image Landsat/Copernicus) (**b**) Geological map of the northern Chinese Tianshan Mountains and the southern Junggar Basin (modified from^26^). The red star indicates the working area, also known as the Anjihaihe section.

The studied section of the Anjihaihe Formation is located to the north of the Chinese Tianshan Mountains, northwest China (Fig. 1a). The region was a basin in the Late Permian and accumulated vast sediments^17,18^. At ca. 24 Ma, the region started to uplift due to the India-Eurasia plate collision and eventually evolved into the northern Tianshan Mountains in the Cenozoic^19–22^. This regional tectonic event also exhumed the Mesozoic to Cenozoic sedimentary strata in this area and formed three rows of foreland thrust-anticline belts in the adjacent southern Junggar Basin (Fig. 1b). The Anjihaihe Formation is exposed in the Huoerguos anticline in the middle belt, which was formed during the Late Pliocene - Early Pleistocene^23–26^. The Anjihaihe Formation conformably overlies the Eocene Ziniquanzi Formation (E_1-2_z), which is composed of purplish-red sandy mudstone and grey conglomerate and is conformably overlain by the Miocene Shawan Formation (N_1_s), which is composed of maroon calcareous cemented sandstone and siltstone. All these strata have a uniform dip angle of 42° towards the north. The paleomagnetic study has revealed that the age of the Anjihaihe Formation is ca. 32.7–23.3 Ma^18,27^, the same time span as the Oligocene. Apatite fission-track analysis of the Cenozoic strata in the Manas section, ~40 km east of the Anjihaihe section, indicates that the burial of these Cenozoic sediments was less than ~2 km^28,29^.

**Figure 2:**
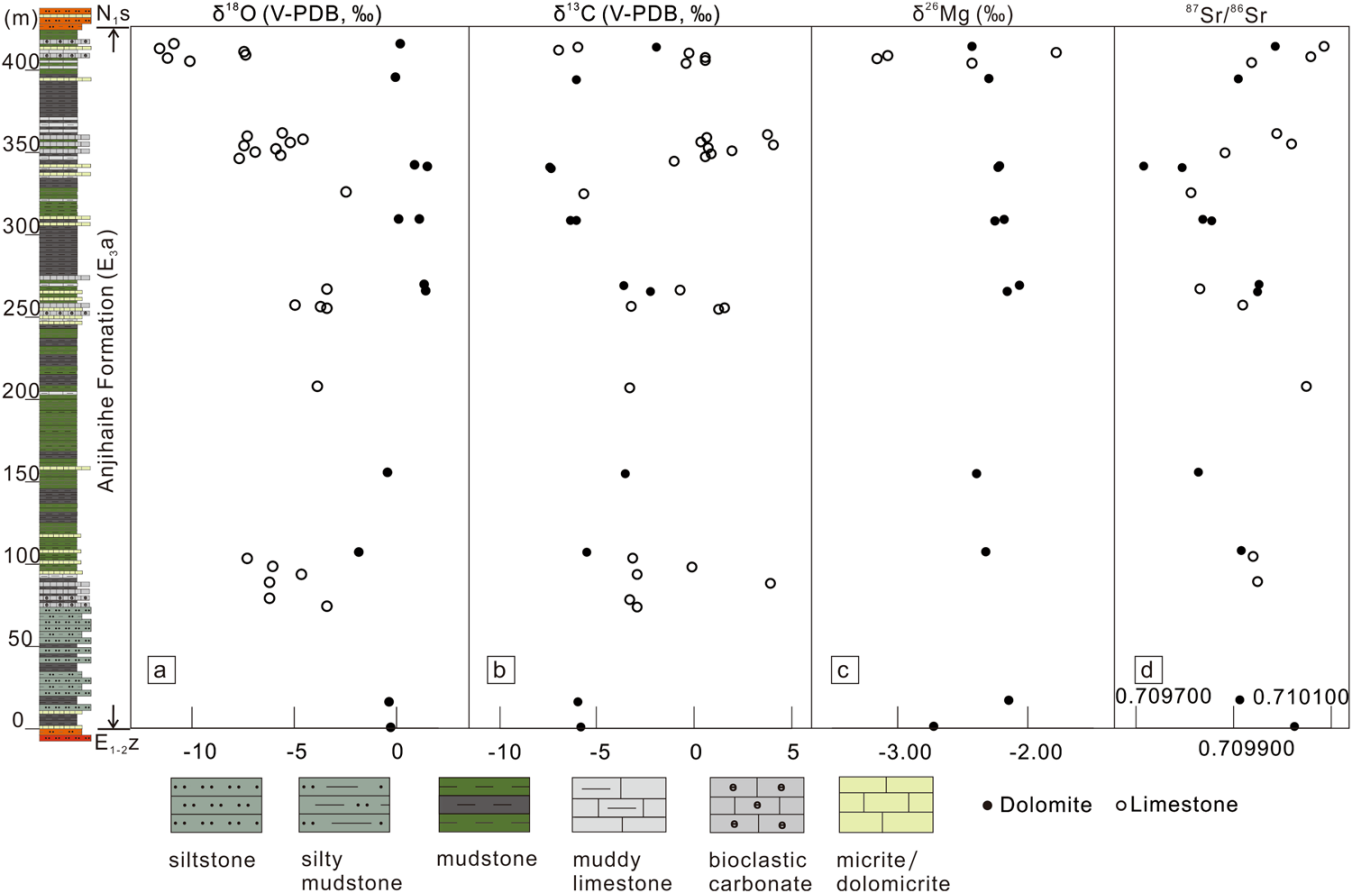
Lithostratigraphic profile of the Anjihaihe section and (**a**) oxygen, (**b**) carbon, (**c**) magnesium, and (**d**) strontium isotopic results of the Anjihaihe dolomite and limestone. The solid circles represent the dolomite samples and the open circles represent the limestone samples.

Sediments in the Anjihaihe Formation are mainly thin-layered, grey-green to dark-green siltstone and silty mudstone interlayered with thin- to medium-bedded carbonate layers, including grey micrite, coarse bioclastic limestone, and white-grey dolomite. The Anjihaihe sediments show consistent typical semi-deep to deep lacustrine facies without evident changes throughout the entire section. In contrast, the overlying Shawan Formation shows a distinctive change in sedimentary conditions to an exposed environment illustrated by the dramatic lithological change from dark green mudstone to maroon sandstone. Although limestone layers also occurred in the underlying Ziniquanzi Formation and the overlying Shawan Formation, the dolomite layers are only found in the Anjihaihe lacustrine sediments. These dolomite layers are mostly 10–20 cm thick with a white-grey chalky texture and are interbedded with lacustrine dark mudstone or limestone layers (Fig. 2).

## Results

The oxygen, carbon, magnesium and strontium isotopes results, along with the Anjihaihe lithostratigraphic profile (also see Supplementary Table S1, S2) are shown in Fig. 2. The δ^13^C values show similar ranges for dolomite (−7.4‰ to −1.9‰; average: −5.1‰) and limestone (−6.9‰ to +4.1‰; average: −0.7‰). In contrast, the δ^18^O value shows a small range from −1.9‰ to +1.5‰ (average: +0.3‰) for dolomite but a much larger range with lower values from −11.6‰ to −2.5‰ (average of −6.3‰) for limestone. The δ^26^Mg values show a limited range from −2.73‰ to −2.07‰ with an average of −2.29‰ for the dolomite beds but a much larger range between −3.16‰ and −1.78‰ with an average of −2.61‰ for the limestone beds. The ^87^Sr/^86^Sr values of dolomite and limestone show similar ranges, from 0.709715 to 0.710024 with an average of 0.709889 for dolomite and 0.709813 to 0.710085 with an average of 0.709956 for limestone.

## Discussion

### Genesis of dolomite

Several features of the Anjihaihe dolomites, such as microcrystals, low Mg/Ca ratios and low degrees of the lattice order, are similar to these of syndepositional nonstoichiometric lacustrine dolomicrite^16^ (see Supplementary Fig. S1). However, unlike most modern lacustrine dolomites^30–36^, neither bacterial effects nor evaporite minerals have been found in the Anjihaihe Formation. This complicates the explanation of the genesis of the Anjihaihe dolomite.

Previous studies suggest that the Anjihaihe dolomite was produced by dolomitization on precursor micrite at low temperatures, based on evidence from SEM observation^16^. This can be verified by our oxygen isotopic results. In the studied section of the Anjihaihe Formation, the limestone beds show relatively stable δ^18^O values from −7.7‰ to −2.5‰ in the lower section but steadily decrease to −11.6‰ toward the top of the section (Fig. 2a). This decreasing trend is consistent with the topographic uplift in the region, which should have induced a progressive decrease in δ^18^O of the meteoric water-dominated lake water, in which the limestones were precipitated. The preservation of the climatic trend in the limestone beds suggests that these limestone beds have not been modified by diagenesis and later dolomitization fluid. In contrast, the δ^18^O values of the dolomite beds do not show such a trend, but display relatively uniform values around 0‰ (−1.9‰ to +1.5‰; average: +0.3‰), which indicates that the primary oxygen isotope compositions of limestones have been completely overprinted by dolomitization from a single fluid rather than multiple episodes of fluids. The high δ^18^O values of dolomites, which are similar to those of carbonate deposited in modern and Oligocene oceans^37,38^, requires a high δ^18^O value of the dolomitization fluid and a large oxygen isotope fractionation, which indicate a low-temperature condition of dolomitization.

Similarly, Mg isotope compositions of limestone show large variation (from −3.16‰ to −1.78‰ in a single bed; Fig. 2c), which is believed to reflect the primary Mg isotopic variation in the lacustrine depositional environment. In contrast, the δ^26^Mg of the dolomite beds show relatively uniform values around −2.3‰ (−2.73‰ to −2.07‰). This again indicates that all the dolomite beds obtained their Mg from a single fluid rather than multiple episodes of fluids. On the other hand, different types of dolomite samples from Europe and North America shows that marine evaporative sabkha dolomites have an average δ^26^Mg of −2.11‰ while the lacustrine and palustrine dolomites have an average δ^26^Mg of −1.25‰^39,40^. The distinct δ^26^Mg between marine and continental dolomite indicates different Mg sources. The similar δ^26^Mg of the Anjihaihe dolomite to those of the marine evaporative dolomites may reflect a seawater influence during its dolomitization.

The carbon and strontium isotope compositions of the dolomite beds, however, show no obvious difference to those of their contacting limestone beds (Fig. 2b,d). The overall variations of δ^13^C values and ^87^Sr/^86^Sr ratios of dolomite follow the variation patterns of the limestone beds, which indicate that the dolomites inherited the carbon and strontium isotopic signatures of their precursor limestones. This implies that the dolomitization fluid was relatively poor in carbon and strontium (compared with those in the limestone) and thus incapable to alter the primary isotopic signatures of carbon and strontium during dolomitization.

In summary, the dolomitization fluid was characterized by high δ^18^O, low δ^26^Mg, low temperature and poor carbon and Sr content. These features are consistent with surface seawater rather than deep brines. Seawater generally has a high δ^18^O value (higher than most of the meteoric waters), a δ^26^Mg value of −0.82‰^39–42^, anda low Sr/Mg ratio^43–45^ (ca 1.6 × 10^−3^). Whereas reactivated old deep brines would be expected to have (1) relatively high strontium content as a result of long-time reaction with the host rock during their burial, and (2) relatively high temperatures because of the heating by tectonic activity.The possibility of deep brines as the fluid source can be also excluded by the absence of dolomitization on the limestone in the underlying Ziniquanzi Formation.

The dolomite layers in the Anjihaihe Formation occur throughout the entire section. These dolomite layers share a similarity in the SEM morphology and XRD spectrum (see Supplementary Fig. S1), yet no stratigraphic sequence pattern was observed in any isotope systems studied here. This suggests that the dolomite layers were not formed due to the burial process, but because of dolomitization by the incursion of seawater. Additionally, the dolomite beds only occurred in the Anjihaihe Formation, and no dolomite was found in the overlying formations, suggesting that the dolomitization time should be earlier than the deposition of the Shawan Formation when the Anjihaihe Formation was just deposited and still sat near the surface in the late Oligocene.

Given the identical petrographic feature and geochemical signatures in multiple isotope systems throughout the large number (>20) of dolomite layers, we consider the dolomitization of these layers could have operated by one episodic incursion of seawater. The seawater could penetrate down to several hundred meters below the surface along some inclined fault. Depending on the connectivity of the faulting system to the limestone layers and the permeability of individual layers, the seawater could then migrate horizontally along some (but not all) limestone layers, which induced the dolomitization of these layers. Although we cannot completely exclude the possibility of multiple seawater incursion events, we speculate that the dolomites in the Anjihaihe Formation are less likely to be syngenetic or para-syngenetic, i.e., formed by dolomitization on surface by repeated transient mixture of lacustrine freshwater and seawater. This is supported by two lines of evidence. First, there are about 20 dolomite layers in the Anjihaihe Formation. A multiple-episode model would require about 20 times of seawater incursion. This high frequency of seawater incursion would suggest a drastic climate fluctuation or a close distance between the ocean and the Anjihaihe lake, which contradicts the sediments evidence in the Tarim Basin^5,6^. Second, multiple incursion would have a better chance to leave depositional evidence along the seaway. However, no marine sediments from the Oligocene or the Miocene have been found in the study area. Therefore, one-time transgression may be a more probable interpretation.

### The incursion of the Paratethys seawater

In the late Oligocene, the only ocean that could possibly provide seawater to the study region was the Paratethys Sea in the west. Previous studies suggest that the eastern margin of the Paratethys Sea during the late Oligocene was around 70°E^13^. However, the geochemical records in the Anjihaihe dolomites imply that the Paratethys seawater might have invaded into the southern part of the Junggar Basin in the late Oligocene. In fact, different studies show that carbonates from the Central Paratethys during Miocene have similar δ^18^O as that of the Anjihaihe dolomite, which reflect the oxygen isotope composition of the Paratethys seawater^46–48^. Moreover, a paleo-valley system existed among the Tianshan Mountains during the Late Cretaceous to the early Miocene before the uplift of the Tianshan Mountains^49^, which could have connected the Junggar Basin to the Paratethys Sea, even just intermittently.

**Figure 3:**
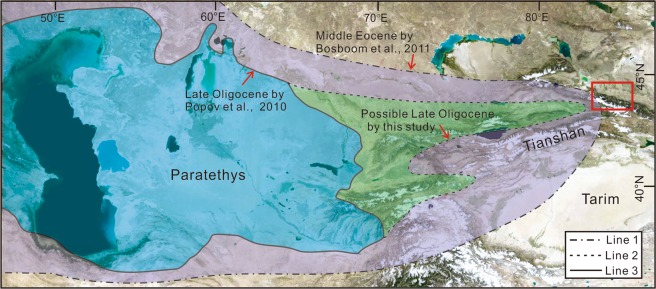
The retreat scheme of the Eastern Paratethys in the central Asian during the Oligocene.The satellite image is from Google Earth (map data: Google, data SIO, NOAA, U.S. Navy, NGA, GEBCO; Image Landsat/Copernicus). Line 1 indicates the possible distribution of the Paratethys in the Middle Eocene by^6^. Line 2 indicates the possible distribution of the Paratethys in the Late Oligocene by our research. Line 3 indicates the possible Paratethys in the Late Oligocene by^13^. The red box indicates the location of the northern Tianshan Mountains and southern part of the Junggar Basin.

During the Oligocene, the westward retreat of the Paratethys Sea was likely controlled by the coeval tectonic activities in the Pamir Mountain and the northern Tianshan Mountains due to the Indian-Eurasian plate collision in the Cenozoic. Because these regions were uplifted heterogeneously^19–22,28^, the retreat might not maintain the same pace in its eastern coastline (Fig. 3). Especially at the northeast part, because the uplift of the northern Tianshan Mountains did not occur until around 24 Ma^19–22,28^, the Paratethys Sea could still be able to reach the northern Tianshan area in the late Oligocene. In addition, the Anjihaihe Formation was not exhumed to the west of the Anjihaihe section, neither was contemporary dolomite reported in this region. On the east side, several sections, where the Anjihaihe Formation was exposed, do not contain dolomites either. This suggests that the sea incursion could have only reached the study area but no further to the east or the west and that the incursion might have come from the south (Fig. 3).

Marine sedimentary evidence show that the Paratethys Sea still existed in the west and southwest part of the Tarim Basin^6^ in the Middle Eocene (Fig. 3 Line 1) but had retreated to the west of the Chinese Tianshan Mountains by the Late Oligocene^13^ (Fig. 3 Line3). The late Oligocene marine sedimentary record in the Chinese Tianshan area is however rare. This may be because this kind of incursion was short-lived so that the sedimentary record would be scarce, or, if there was any, have been eroded during the uplift of the northern Tianshan Mountains and the northward indentation of the Pamirs in the Neogene. Nonetheless, the stable isotope results of the Anjihaihe dolomite suggest that this retreat might not be so fast. Based on the different uplift time in this region^19–22,28^, we built up a model for the possible approximate extent of the Paratethys in the Late Oligocene (Fig. 3 Line 2).

Previous numerical experiments suggested that the initial retreat of the Paratethys westward could have similar effects as the uplift of the Tibetan Plateau on the formation of the Asian monsoon but the Paratethys Sea retreat to the Turan Plate during the Late Oligocene/Earliest Miocene was the turning point for the climate in northwest China, from “planetary-wind-dominant type” to “monsoon dominant type”^3^. The retreat of the Paratethys was thought to result in the loss of moisture source and increase the land-sea thermal contrasts, which would facilitate the onset of the Asian monsoon in the Cenozoic^1,3,5,6^. However, the isotopic records of the Anjihaihe Formation in the Junggar Basin suggest that the Paratethys Sea could have incurred into the southern Junggar Basin,which indicates that the westward retreat of the Paratethys may not be as quick as previously thought. Subsequently, its role in facilitating the onset and evolution of the Asian monsoon needs revaluation. Accordingly, the uplift of the Tibet Plateau and global cooling could be the main mechanisms for the initiation of the Asian monsoon.

## Conclusions

Comparison of carbon, oxygen, magnesium, and strontium isotope compositions between the dolomite and unaltered precursor limestone in the Oligocene lacustrine Anjihaihe Formation in the Junggar Basin indicate that the dolomitization fluid was dominated by seawater and had a low temperature. This seawater was likely from the Paratethys Sea, which suggests that, by the late Oligocene, the Paratethys Sea remained coverage close to the Tianshan Mountain area. This indicates a much larger Oligocene Paratethys Sea than previously thought and subsequently implies less influence of the Paratethys Sea on the onset and evolution of the Asian monsoon climate.

## Materials and methods

Carbonates samples, including dolomite and limestone, from the Anjihaihe Formation, were collected along a cross-section. Each sample was then polished into thin sections for SEM study and crushed into fine powders (less than 200 mesh) for XRD, carbon, oxygen, strontium, and magnesium isotopic measurements.

Oxygen and carbon isotope analysis were carried out in the Analytical Laboratory of the Beijing Research Institute of Uranium Geology by a Thermo FinniganGasBench II linked to a Finnigan MAT 253 mass spectrometer. Two Chinese carbonate standards (i.e., GBW04416 with δ^13^C = 1.61 ± 0.03‰ and δ^18^O = −11.59 ± 0.11‰ and GBW04417 with δ^13^C = −6.06 ± 0.06‰ and δ^18^O = −24.12 ± 0.19‰) were added to every 5 samples during analysis. The results were reported relative to the Vienna Pee Dee Belemnite standard (V-PDB), with uncertainties better than ±0.1‰ (2σ) for δ^13^C and ±0.2‰ (2σ) for δ^18^O.

For magnesium isotope analysis, magnesium was extracted and purified at Peking University following^50–53^. Powder samples were loaded with 5 mL of 1 N acetic acid in a tube, which was then placed in an ultrasonic bath for two days to ensure complete dissolution of carbonate. Ca was removed using column #1 (loaded with 1.8 mL of Bio-Rad 200–400 mesh AG50W-X12 resin). The Mg fraction was collected in 5 mL of 12 N HCl whereas Ca was retained in the resin. Then Cr, Al, Fe, Na, and K were removed by column #2 (loaded with 0.5 ml of Bio-Rad 200–400 mesh AG50W-X12 resin). The magnesium isotopic ratios were then measured at the Isotope Laboratory of China University of Geosciences (Beijing) using a Thermo Scientific Neptune Plus high-resolution multi-collector inductively coupled plasma mass spectrometer (MC-ICP-MS). δ^26^Mg and δ^25^Mg were reported relative to the DSM-3 standard^54^. The internal precision was determined by repeated runs (three times) of the same sample solution during a single analytical session and was better than 0.1‰ (2σ). The external precision was determined by measurements of the GSB-Mg and USGS basalt standards (BCR-2 and BHVO-2). The δ^26^Mg value of GSB-Mg (−2.03 ± 0.03‰; 2σ), BCR-2 (−0.16 ± 0.05‰; 2σ) and BHVO-2 (−0.25 ± 0.04‰; 2σ) are consistent with the laboratory recommended values or previously published data^49–52,55^.

The strontium isotopic analysis was carried out at Peking University. 200 mg of each powdered samples were resolved with 2 ml 2.5 N HCl in 10 ml beakers and sit for 45 minutes for total dissolution. The samples were evaporated to dry on a hot plate at 120 °C. Add 1 ml 2.5N HCl and dry them on a hot plate at 120 °C again. After the samples were cooled down, add 1.2 ml 2.5N HCl and sit overnight. Move the samples from beakers to tubes and centrifuge for 10 min at 3000 rpm. After loading the supernatants in cation-exchange columns filled with AG 50W X12 (200–400 mesh)resin, rinse them with 0.5 ml 2.5N HCl and 7.5 ml 5N HCl. Sr was then collected using 3 ml 5N HCl. The strontium isotope values were determined using Nu Plasma II MC-ICP-MS. The external precision was determined by measurements of the BCR-2 and the accuracy is better than ±0.000020.

## Supplementary information

Supplementary information.
