# Supplementary material for: Oligocene incursion of the Paratethys seawater to the Junggar Basin, NW China: insight from multiple isotopic analysis of carbonate
Source: Sci Rep. 2020 Apr 20;10:6601. doi: 10.1038/s41598-020-63609-0 (PMC7170927; doi:10.1038/s41598-020-63609-0)
Supplement: Supplementary file 1 — Supplementary information. [file 41598_2020_63609_MOESM1_ESM.pdf]

Supplementary Material

Oligocene incursion of the Paratethys seawater to the Junggar Basin, NW China: insight from multiple isotopic analysis of carbonate

Qian Li[a,b], Long Li[b], Yuanyuan Zhang[a], Zhaojie Guo[a*]

[a]Key Laboratory of Orogenic Belts and Crustal Evolution, School of Earth and Space Science, Peking University, Beijing 100871, PR China

[b]Department of Earth and Atmospheric Sciences, University of Alberta, Edmonton, AB, Canada

Supplementary Table S1. Carbon, oxygen, magnesium, and strontium isotopic results of the Anjihaihe dolomite.

Sample No.	Position/m	$\delta^{13}C_{V\text{-}PDB}$/‰	$\delta^{18}O_{V\text{-}PDB}$/‰	$\delta^{26}Mg$/‰	2σ (Mg)	$^{87}Sr/^{86}Sr$
15AJ1-9	420	-1.9	0.2	-2.44	0.03	0.709985
15AJ1-13	400	-6.0	-0.1	-2.31	0.04	0.709909
15AJ3-1	346	-7.4	0.9	-2.22	0.02	0.709715
15AJ3-2	345	-7.3	1.5	-2.23	0.02	0.709792
15AJ4-1	313	-6.3	0.1	-2.19	0.02	0.709835
15AJ4-2	312.8	-6.0	1.1	-2.26	0.01	0.709853
15AJ4-3	273.2	-3.6	1.3	-2.07	0.05	0.709953
15AJ5-2	269	-2.2	1.4	-2.16	0.03	0.709949
15AJ7-1	157	-3.5	-0.5	-2.39	0.03	0.709826
15AJ8-2	109	-5.5	-1.9	-2.32	0.03	0.709915
15AJ10-1	17	-5.9	-0.4	-2.16	0.02	0.709912
15AJ10-3	1	-5.8	-0.3	-2.73	0.02	0.710024
Max.		-1.9	1.5	-2.07	0.05	0.710024
Min.		-7.4	-1.9	-2.73	0.01	0.709715
Ave.		-5.1	0.3	-2.29	0.03	0.709889

* Corresponding author. Email address: zjguo@pku.edu.cn (Z. Guo)

Supplementary Table S2. Carbon, oxygen, magnesium, and strontium isotopic results of the Anjihaihe limestone.

Sample No.	Position/m	$\delta^{13}C_{V\text{-}PDB}$/‰	$\delta^{18}O_{V\text{-}PDB}$/‰	$\delta^{26}Mg$/‰	2σ (Mg)	$^{87}Sr/^{86}Sr$
14AJ13-1	420	-5.9	-10.9			0.710085
14AJ13-2	418	-6.9	-11.6			
14AJ14-1	416	-0.2	-7.5	-1.78	0.02	
14AJ14-2	414	0.6	-7.4	-3.08	0.06	0.710058
14AJ14-4	412	0.6	-11.2	-3.16	0.05	
14AJ14-5	410	-0.4	-10.1	-2.43	0.02	0.709938
14AJ15-1	366	3.8	-5.6			0.709990
14AJ15-2	364	0.7	-7.3			
14AJ15-3	362	0.4	-4.6			
14AJ15-5	360	4.1	-5.2			0.710018
14AJ15-6	358	0.8	-7.5			
14AJ15-7	356	2.0	-5.9			
14AJ15-8	354	0.9	-6.9			0.709881
14AJ15-9	352	0.6	-5.7			
14AJ15-15	350	-1.0	-7.7			
14AJ16-1	330	-5.6	-2.5			0.709813
15AJ5-1	270	-0.7	-3.4			0.709831
14AJ17-2	260	-3.2	-5.0			0.709919
15AJ5-4	259	1.6	-3.7			
15AJ5-5	258	1.3	-3.4			
14AJ19-1	210	-3.3	-3.9			0.710048
14AJ12-6	105	-3.1	-7.3			0.709939
14AJ12-5	100	-0.1	-6.1			
14AJ12-4	95	-2.9	-4.7			
14AJ12-3	90	4.0	-6.2			0.709950
14AJ20-2B	80	-3.3	-6.2			
14AJ20-3	75	-2.9	-3.4			
Max.		4.1	-2.5	-1.78		0.710085
Min.		-6.9	-11.6	-3.16		0.709813
Ave.		-0.7	-6.3	-2.61		0.709956

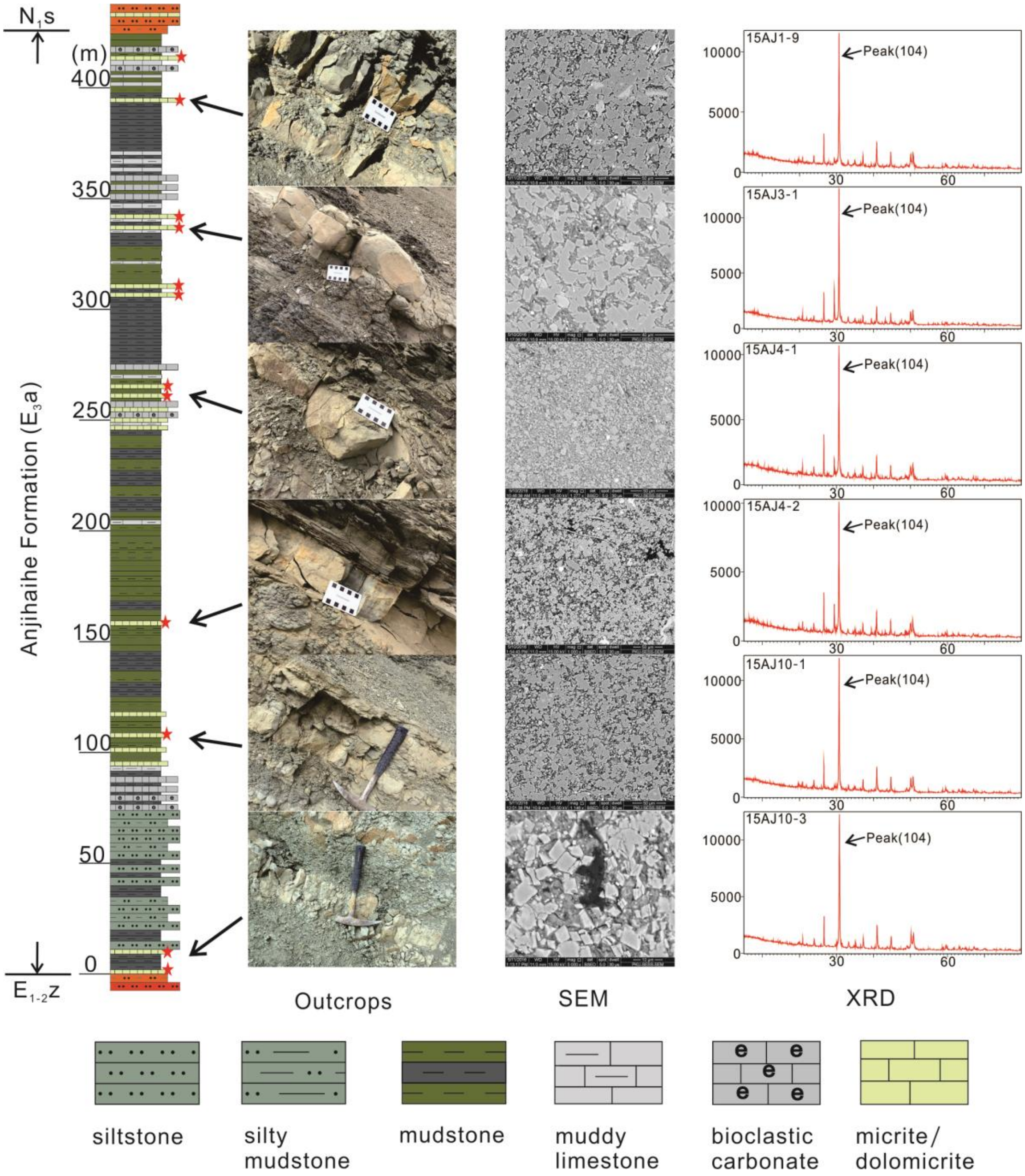

Supplementary Figure S1. Lithostratigraphic profile of the Anjihaihe section, pictures of representative dolomite samples and their corresponding SEM morphology and XRD spectra. The outcrop pictures show that the Anjihaihe dolomite beds are interlayered with the thin-layered, grey-green to dark-green siltstone and silty mudstone beds. Red stars on the lithostratigraphic profile indicate the sample layers. SEM pictures show that these dolomite samples are subhedral to euhedral microcrystalline dolomicrite. XRD spectra show that dolomite crystals are the main mineral in the samples. Peak (104) is the

characteristic peak of dolomite. Samples in different stratigraphic levels have a similar appearance, SEM morphology, and XRD spectra.
